# Food insecurity is associated with low diet quality and unhealthy cooking and eating habits in Iranian women

**DOI:** 10.1186/s41043-024-00533-3

**Published:** 2024-03-14

**Authors:** Ali Kohanmoo, Maral Hashemzadeh, Maryam Teymouri, Morteza Zare, Masoumeh Akhlaghi

**Affiliations:** 1 Department of Community Nutrition, School of Nutrition and Food Sciences, Shiraz University of Medical Sciences, Shiraz, Iran; 2 Department of Clinical Nutrition, School of Nutrition and Food Sciences, Shiraz University of Medical Sciences, Shiraz, Iran

**Keywords:** Food insecurity, Diet, Dietary pattern, Eating habits

## Abstract

**Background:**

Food insecurity affects diet and nutrition intakes. We explored the relationship between food insecurity and dietary intakes in a group of Iranian women.

**Methods:**

The cross-sectional study was performed on 190 healthy females aged 20–55 years attending primary healthcare centers in Shiraz. Food insecurity was evaluated by Household Food Insecurity Access Scale, which is a validated tool for assessing food insecurity in developing countries. Diet was assessed using a food frequency questionnaire. The association of dietary patterns and food insecurity was assessed by linear regression.

**Results:**

Assessment of dietary intakes revealed that consumption of red meat, poultry, fish, dairy, fruits, non-starchy vegetables, and nuts decreased whereas that of grains, processed meats, potato, and sugary foods increased with increasing food insecurity. Among nutrients, carbohydrates, fiber, vitamin A, vitamin C, folic acid, potassium, calcium, and magnesium decreased while fat and sodium increased as food insecurity increased. Three major dietary patterns were detected. Healthy dietary patterns showed inverse associations with food insecurity in the crude (β = -0.422 and − 0.435, *P* < 0.001) and adjusted (adjusted for age, marital status, and educational level) (β = -0.475 and − 0.341, *P* < 0.001) models of regression analysis but unhealthy pattern did not show an association with food insecurity. Compared to food secure participants, a higher percentage of food insecure individuals indicated unhealthy eating habits, such as skipping breakfast, lower snack ingestion, more fast and fried food consumption, and using unhealthy cooking methods.

**Conclusion:**

Overall, this study showed that food insecurity was associated with less healthy diet and unhealthy cooking and eating habits.

**Supplementary Information:**

The online version contains supplementary material available at 10.1186/s41043-024-00533-3.

## Introduction

Food insecurity is a common nutrition problem around the world [1]. According to the reports of Food and Agriculture Organization of the United Nations, nearly 30% of the world population suffered from moderate to severe food insecurity in 2021 [2]. In Iran, moderate and severe food insecurity was estimated between 40 and 60% [2].

Food insecurity has a close link with negative health outcomes particularly chronic metabolic diseases and mental problems [3, 4]. It has been correlated with increased mortality [5] even among infants [6]. Food insecurity has been related to stress, anxiety, and depression during pregnancy, and also birth defects, neonatal mortality and the early introduction of animal milk to infant’s diet [7]. In children, food insecurity has been associated with higher school absenteeism and lower academic grades [8]. In college students, this association was proved to be mediated by adverse effect of food insecurity on students’ psychosocial health [9]. Food insecurity increases costs of health services, reduces work efficiency and productivity, and harms the economy [10]. Food insecure adults are estimated to have annual health care costs of $1,834 more than food secure adults [11]. For children, the difference was less and about $80.

The link between food insecurity and poor health status may be explained by poor diet quality. Insufficient income is a key barrier for having a healthy diet [12]. In fact, when there is no financial constraint, people are able to design and afford a food plan containing a wide variety of foods from different food groups [13, 14]. When financial resources are limited, people have to diminish food costs by eliminating expensive food items, such as fruit and vegetables, from the diet, resulting in a less diverse and thus less healthy diet [15]. In such cases, nutrient-dense foods are eliminated and energy-dense foods are substituted, resulting in a shift from a healthy diet to an unhealthy regimen [16].

In recent years and concomitant with worsening economic conditions in COVID-19 pandemic, the number of investigations examining the relationship of food insecurity and diet quality has dramatically increased. The majority of investigations have declared an inverse association between food insecurity and diet quality. As an example, in a large group of older adults, food insecurity was associated with lower scores on the Healthy Eating Index, the Alternate Healthy Eating Index-2010, and the Mediterranean Diet Score [17]. Also, a study on emerging US adults showed that food insecurity was associated with poorer diet quality (e.g., less vegetables and whole grains, more sugar-sweetened beverages, and added sugar), lower home availability of healthy foods, skipping breakfast, frequently eating at fast-food restaurants, binge eating and drinking, and substance use [18].

However, not all studies have shown such association. For instance, a study on low-income adults revealed associations between food insecurity and compromised diet quality in winter and during the COVID-19 period but not during fall nor during the pre-COVID-19 months [19]. Also, in a large cross-sectional study in Canada, the intake of carbohydrates, total sugar, fat, and saturated fat, the main items of an unhealthy diet, did not differ by food insecurity status [20]. Due to controversies in the literature and because the association between food insecurity and diet quality is assumed to vary by race/ethnicity [21], we aimed to examine the association of food insecurity with diet quality and eating habits in healthy Iranian women. As previous studies have examined the relationship of food insecurity with dietary pattern using “a priori” approach, in this study, the association of food insecurity with “a posteriori” dietary patterns was investigated.

## Methods

### Study design

The cross-sectional study was performed in spring and summer of 2016 in Shiraz. A sample size of 190 was calculated using a 16.1% prevalence rate for food insecurity as reported by previous reports [22], a confidence interval of 95%, and 5% margin of error.

### Subjects

Participants were chosen from women visiting primary healthcare centers in all nine municipal districts of Shiraz. Selection of primary healthcare centers was done by random sampling method, in which 20 out of 38 centers located in Shiraz were randomly selected. Then, an average of 10 women from each center were selected by convenience sampling. The participants were clients who visited the centers on sampling days for various healthcare purposes, such as child’ growth monitoring and vaccination, visiting a physician, and women’s health care.

Inclusion criteria were as follows: healthy women aged 20 to 55 years without special medical conditions, such as cancer, chronic metabolic diseases, thyroid abnormalities, renal or hepatic disorders, eating disorders, pregnancy, and lactation. They also were not on special diets. The purpose and procedure of the research was explained to the participants, and if they agreed, a signed consent form was obtained. The project was approved by the ethics committee of Shiraz University of Medical Sciences (project number: 93-7271).

### Food insecurity

Food insecurity was evaluated by Household Food Insecurity Access Scale (HFIAS) which is approved to examine food insecurity in developing countries [23]. The questionnaire is composed of 9 items and has been translated and validated for applying in the Iranian community [24]. The HFIAS categorizes food insecurity into four levels of food secure (0–1), and mild (2–8), moderate (9–16), and severe (17–27) food insecure [25].

### Dietary assessment

Dietary assessments were performed by a validated semi-quantitative food frequency questionnaire (FFQ) [26]. The FFQ consisted of a list of food items with serving sizes commonly consumed by Iranians. Participants were asked to report the frequency of foods consumed in designated serving sizes during the past one year on a daily, weekly, or monthly basis. Consumed foods were then converted to grams and their nutrient composition was determined by modified Nutritionist IV version 3.5.2 with a nutrient database based on the US Department of Agriculture (USDA) food composition tables modified for Iranian foods.

### Eating habits

Eating habits were determined by questioning about the frequency of main meals and snacks as well as fried and fast foods, techniques of cooking including that used for cooking rice, and the habit of consuming tea with or immediately after meals. The last two items related to cooking and eating habits of Iranians [27, 28].

### Statistical analysis

Data were analyzed by SPSS version 19 (SPSS Inc., Chicago, IL, USA). Dietary patterns were identified using the factor analysis method [29]. Some FFQ items had missing values (the number of missing values for each food group is presented as supplemental materials). Before extracting dietary patterns, the missing values were replaced based on multiple imputation method using available data. Missing-at-random assumption was used to generate 10 sets of imputed data, the pooled of which was used in the analysis as suggested by Rubin [30].

Food items of FFQ were categorized into 14 groups and dietary patterns were extracted by principal component factor analysis with varimax rotation on the 14 food groups. Components with eigenvalues &amp;gt; 1 were retained. In the scree plot, three major dietary patterns were located before a clear inflection. The Bartlett factor of the dietary patterns was used for statistical analysis. Higher Bartlett scores indicated higher adherence.

The association of food insecurity levels with food and nutrient intake and eating habits was examined with one-way analysis of variance (ANOVA). Due to low number of individuals in severe food insecurity (*n* = 2), participants with moderate and severe food insecurity were combined in a category named as moderate/severe food insecurity. Therefore, the levels of food insecurity were set as food secure, mild food insecure and moderate/severe food insecure. Before analysis, the normality of the data was assessed with Kolmogorov-Smirnov test, and Kruskal–Wallis test was used when the null hypothesis for data normality was rejected.

Linear regression was used to examine the association of dietary patterns with food insecurity. The normality of the residuals was assessed with histograms which showed normal distribution. Since there were differences in demographic characteristics of the participants and considering that such features may affect food choices and dietary patterns [31–33], age, marital status, and educational level were used as covariates in the adjusted model to see if differences in these variables between individuals influence food insecurity-dietary pattern relationships. Statistical analysis was set at *P* < 0.05.

## Results

**Fig. 1:**
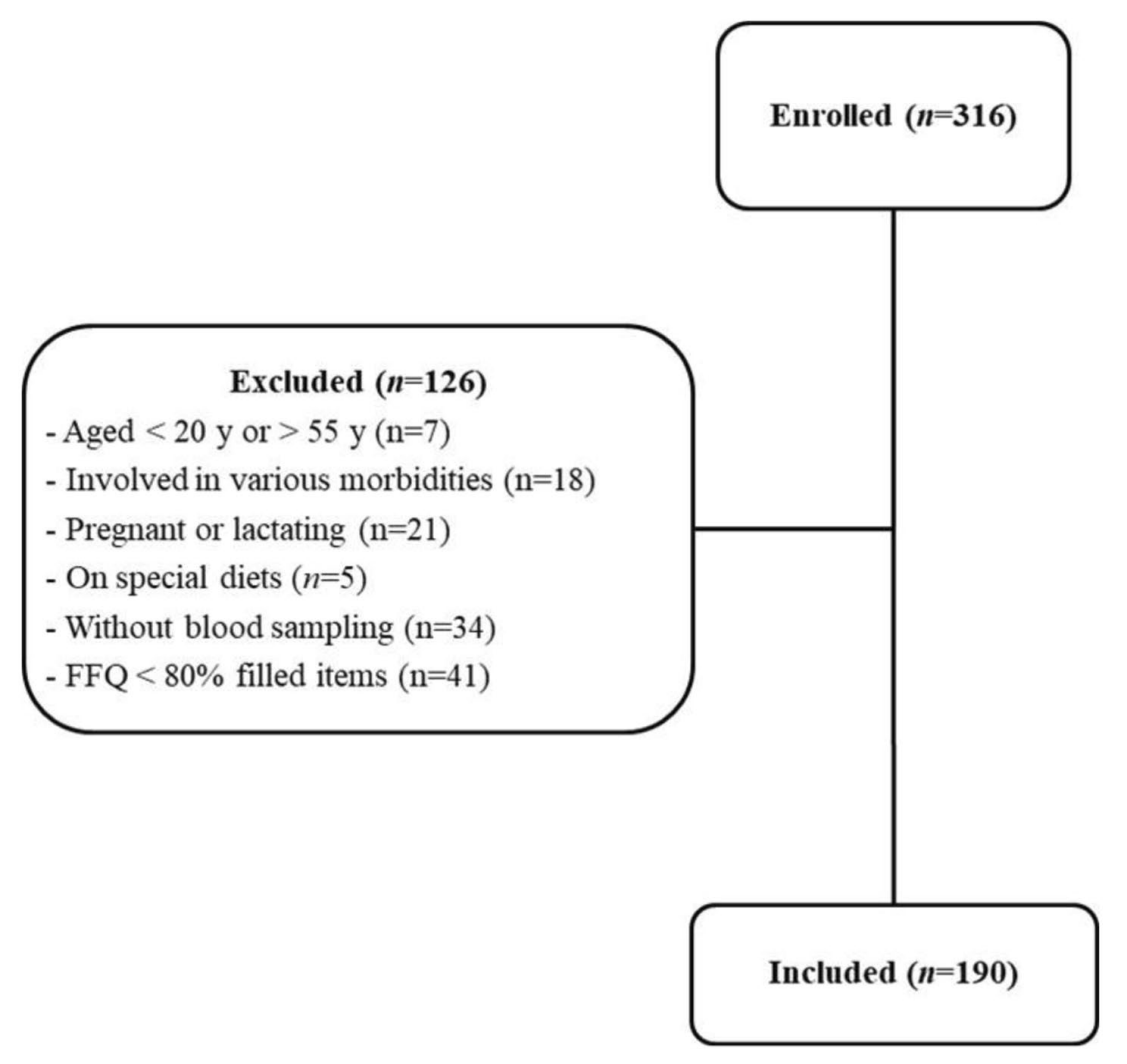
Flowchart of the study participants

The flow diagram of the study enrolment is illustrated in Fig. 1. Out of a total of 275 women who accepted to participate in the study, 126 were excluded according to the inclusion/exclusion criteria, and 190 women included. They aged on average 35.2 ± 8.2 years and had a mean body mass index (BMI) of 27.7 ± 5.4 kg/m^2^. According to the classification described in the Methods, 81 women (42.6%) were food secure, 77 (40.5%) were mild food insecure, 30 (15.8%) were moderately food insecure, and 2 (1.1%) were severely food insecure. General characteristics of the participants based on the levels of food insecurity are presented in Table 1. Participants in different food insecurity levels did not differ in age and marital status but the educational level of the participants as well as that of the household head was higher in food secure women (*P* < 0.001).

**Table 1:** General characteristics of the participants based on food security levels^1,2^

	Secure (n = 81)	Mild insecure (n = 77)	Moderate/severe insecure (n = 32)
Age, y	34.9 ± 8.5	35.6 ± 8.5	35.2 ± 6.7
Body mass index, kg/m^2^	25.2 ± 3.9	28.1 ± 5.1	33.1 ± 5.4
Education level, n (%)			
School College	38 (46.9) 43 (53.1)	68 (88.3) 9 (11.7)	32 (100) 0
Marital status, n (%)			
Single Married	12 (14.8) 69 (85.2)	11 (14.3) 66 (85.7)	9 (28.1) 23 (71.9)
Household size, n (%)			
1–2 3–4 > 4	17 (21.0) 54 (66.7) 10 (12.3)	14 (18.2) 52 (67.5) 11 (14.3)	5 (15.6) 18 (56.3) 9 (28.1)
Family head education, n (%)			
School College	46 (56.8) 35 (43.2)	63 (81.8) 14 (18.2)	32 (100) 0

^1^ Scores of food security levels are as follows: food secure (0–1), mild food-insecure (2–8), moderate/severe food-insecure (9–27). ^2^ Data are expressed as n (%) or means ± SD

Assessment of dietary intakes revealed that consumption of red meat, poultry, fish, dairy, fruits, non-starchy vegetables, and nuts decreased whereas that of grains, processed meats, potato, and sugary foods increased with increasing food insecurity (*P* < 0.05) (Table 2). Legumes and eggs did not show an association.

**Table 2:** Consumption of food groups (g/day) in different food insecurity levels^1,2^

	Secure (*n* = 81)	Mild insecure (*n* = 77)	Moderate/severe insecure (*n* = 32)	***P*** value^3^
Grains	391.7 ± 175.5	426.6 ± 128.4	433.0 ± 134.1	0.022
Legumes	40.0 ± 27.7	35.2 ± 31.2	34.6 ± 26.3	0.186
Red meat	21.9 ± 20.8	13.0 ± 12.3	6.4 ± 7.5	< 0.001
Poultry	5.6 ± 7.6	3.0 ± 3.7	1.7 ± 2.9	0.001
Fish	14.5 ± 18.8	2.9 ± 3.3	0.93 ± 1.9	< 0.001
Processed meats	12.8 ± 19.6	36.8 ± 36.2	60.0 ± 43.2	< 0.001
Eggs	27.2 ± 16.2	26.1 ± 13.5	30.4 ± 16.0	0.321
Dairy	294.8 ± 180.3	193.6 ± 115.0	169.1 ± 98.2	< 0.001
Fruits	440.0 ± 222.4	268.2 ± 226.3	119.2 ± 115.0	< 0.001
Non-starchy vegetables	404.8 ± 158.6	316.3 ± 141.2	294.6 ± 87.1	< 0.001
Potato	30.8 ± 23.9	36.8 ± 19.6	41.8 ± 21.7	0.018
Nuts	7.4 ± 9.9	5.3 ± 9.6	3.8 ± 6.2	0.001
Sugary foods^4^	116.0 ± 168.0	140.2 ± 87.7	166.0 ± 75.6	< 0.001

^1^ Scores of food security are as follows: food secure (0–1), mild food-insecure (2–8), moderate/severe food-insecure (9–27). ^2^ Data are presented as means ± SD. ^3^*P* value was determined by one-way analysis of variance. ^4^ sugary foods included sugar-sweetened beverages, cakes, biscuits, cookies, confections, candies, ice cream, etc.

Among nutrients, carbohydrates (*P* = 0.039), fiber (*P* < 0.001), vitamin A (*P* < 0.001), vitamin C (*P* < 0.001), folic acid (*P* < 0.001), potassium (*P* < 0.001), calcium (*P* < 0.001), and magnesium (*P* < 0.001) decreased while fat (*P* = 0.002) and sodium (*P* = 0.001) increased as food insecurity increased (Table 3). Energy, protein, cholesterol, iron, and zinc did not show an association.

**Table 3:** Daily nutrient intakes in different food security levels^1,2^

	Secure (*n* = 81)	Mild insecure (*n* = 77)	Moderate/severe insecure (*n* = 32)	***P*** value^3^
Energy (kcal)	2197 ± 537	2156 ± 540	2231 ± 488	0.785
Carbohydrate (g)	348.9 ± 93.2	316.8 ± 80.3	299.0 ± 54.6	0.039
Protein (g)	65.4 ± 16.3	61.1 ± 13.9	63.3 ± 15.7	0.298
Fat (g)	60.0 ± 20.2	71.6 ± 33.7	86.9 ± 34.8	0.002
Cholesterol (g)	199.5 ± 78.8	200.2 ± 83.6	240.0 ± 99.1	0.077
Fiber (g)	27.0 ± 8.9	20.2 ± 7.9	16.9 ± 5.1	< 0.001
Vitamin A (µg RE)	707.4 ± 405.9	516.6 ± 300.3	418.5 ± 276.0	< 0.001
Vitamin C (mg)	319.6 ± 149.5	196.8 ± 123.2	149.7 ± 90.7	< 0.001
Folic acid (µg)	328.9 ± 117.8	239.8 ± 95.9	221.5 ± 84.6	< 0.001
Vitamin B_12_ (µg)	5.1 ± 4.8	3.9 ± 3.5	5.5 ± 6.5	0.017
Sodium (mg)	747 ± 343	1162 ± 742	1411 ± 843	0.001
Potassium (mg)	4148 ± 1391	3054 ± 1130	2735 ± 809	< 0.001
Calcium (mg)	745.7 ± 269.6	582.5 ± 172.5	575.0 ± 190.0	< 0.001
Magnesium (mg)	278.9 ± 75.3	224.2 ± 62.4	212.5 ± 53.5	< 0.001
Iron (mg)	15.1 ± 4.2	14.3 ± 3.6	14.3 ± 3.1	0.730
Zinc (mg)	6.9 ± 1.8	6.3 ± 1.9	6.8 ± 1.9	0.176

^1^ Scores of food security are as follows: food secure (0–1), mild food-insecure (2–8), moderate/severe food-insecure (9–27). ^2^ Data are presented as means ± SD. ^3^ P value was determined by one-way analysis of variance

Three major dietary patterns were detected in our study participants (Table 4). The first pattern contained mainly fruits, red meat, nuts, legumes, and non-starchy vegetables and was low in processed meats (healthy dietary pattern 1). The second pattern composed mainly from fish, chicken, low-fat dairy, and non-starchy vegetables but was low in processed meats, grains, and sugary foods (healthy dietary pattern 2). The third pattern contained potato, high-fat dairy, sugar-containing products, egg, processed meats, and grains (unhealthy pattern).

**Table 4:** Food groups and their factor loadings in the three extracted dietary patterns

Food groups	Healthy dietary pattern 1	Healthy dietary pattern 2	Unhealthy pattern
Fruits	0.833	0.112	-0.223
Red meat	0.704		0.286
Nuts	0.612		
Legumes	0.537		-0.130
Processed meats	-0.464	-0.358	0.436
Fish		0.821	
Chicken	-0.156	0.760	0.142
Low-fat dairy		0.588	-0.158
Non-starchy vegetables	0.405	0.497	
Grains		-0.447	0.433
Potato	-0.128	-0.114	0.571
High-fat dairy	0.108	0.113	0.557
Sugary products		-0.266	0.533
Egg	-0.152		0.446

Healthy dietary patterns showed inverse associations with food insecurity in the crude (β = -0.422 and − 0.435, *P* < 0.001), and adjusted (adjusted for age, marital status, and educational level) (β = -0.475 and − 0.341, *P* < 0.001) model (Table 5). Unhealthy pattern did not show an association with food insecurity.

**Table 5:** Regression analysis assessing the association between levels of food insecurity and major dietary patterns^1^

	Crude model			Adjusted model^2^	
	β	***P*** value		β	***P*** value
Healthy dietary pattern 1	-0.422	< 0.001		-0.475	< 0.001
Healthy dietary pattern 2	-0.435	< 0.001		-0.341	< 0.001
Unhealthy dietary pattern	0.054	0.462		-0.050	0.538

^1^ The associations were examined by linear regression. ^2^ Adjusted for age, marital status, and educational level

Frequency of breakfast, lunch, and snack consumption decreased along with increasing food insecurity (*P* < 0.01). (Table 6). On the contrary, the frequency of fast and fried foods increased as food insecurity increased (*P* ≤ 0.001). Food secure participants used boiling as the main technique of cooking while frying was the main method of cooking among food insecure participants (*P* = 0.001). Although in each category of food insecurity, more women were draining rice than doing it as a pilaf, 96.9% of women in moderate/severe food insecurity level drained rice while 63.0% and 71.4% of women in food secure and mild insecure levels drained it, respectively (*P* = 0.005). Compared to food secure families more women in food insecure categories drunk tea with or immediately after meals (56.2% vs. 25.9%) (*P* = 0.001).

**Table 6:** Eating habits in different food security levels^1,2^

	Secure (n = 81)	Mild insecure (n = 77)	Moderate/severe insecure (n = 32)	***P*** value^3^
Breakfast frequency (n/week)	5.9 ± 2.0	5.3 ± 2.1	3.4 ± 2.5	< 0.001
Lunch frequency (n/week)	6.8 ± 0.7	7.0 ± 0.1	6.7 ± 0.9	0.007
Dinner frequency (n/week)	6.1 ± 1.6	6.0 ± 1.5	5.8 ± 1.4	0.083
Meal frequency (n/day)	2.7 ± 0.4	2.6 ± 0.4	2.3 ± 0.5	< 0.001
Snack frequency (n/day)	1.9 ± 1.0	1.6 ± 1.1	1.2 ± 0.6	0.001
Fried foods (n/week)	2.8 ± 1.7	3.5 ± 1.7	4.1 ± 2.1	0.001
Fast foods (n/week)	1.7 ± 1.4	2.8 ± 2.5	5.2 ± 3.9	< 0.001
Main cooking method				
Boiling	48 (59.3)	23 (29.9)	10 (31.2)	0.001
Frying	33 (40.7)	54 (70.1)	22 (68.8)	
Method of cooking rice				
Pilaf	30 (37.0)	22 (28.6)	1 (3.1)	0.005
Draining	51 (63.0)	55 (71.4)	31 (96.9)	
Tea after meals				
Yes	21 (25.9)	41 (53.3)	18 (56.2)	0.001
No	60 (74.1)	36 (46.7)	14 (43.8)	

^1^ Scores of food security are as follows: food secure (0–1), mild food-insecure (2–8), moderate/severe food-insecure (9–27). ^2^ Data are presented as means ± SD. ^3^*P* value was determined by one-way analysis of variance or chi-square (for the last 3 items)

## Discussion

### Food insecurity and dietary patterns

Results presented here showed an inverse association between healthy dietary patterns and food insecurity. Women in food secure conditions had healthier diet, consumed more fruit and vegetables, low-fat dairy, meats, legumes, and nuts, and had greater intake of fiber, vitamin A, vitamin C, folic acid, calcium, magnesium, and potassium. In contrast, food insecure women consumed processed meats more often and had lower intakes of essential nutrients and higher intake of fat and sodium. These results are in accordance with previous findings that have reported an inverse association between food insecurity and Mediterranean-like dietary pattern in adolescents of Lebanon [34], university students of Greece [35], and adults of Portugal [36]. However, in contrast to Jimenez Rincon et al. [19] who observed the association between food insecurity and diet quality in winter and during COVID-19 pandemic but not in fall nor in pre-COVID times, we observed this association during spring and summer and prior to COVID-19 outbreak. The difference in food insecurity assessment tool and the severity of food insecurity between nations may have caused such controversy.

Various factors influence food choice. These include, but not limited to, food preferences, availability, budget and financial ability, knowledge, and attitude. Financial ability is an absolutely important determinant of food choice. However, it is influenced by factors such as knowledge and food preferences. In our study, participants in food insecure families and also the head of food insecure households had lower levels of education compared to food secure participants. Poor nutritional knowledge as a result of low educational level could act as an additional factor along with budget constraints in selection of unhealthy foods by food insecure individuals and families [37, 38]. In this regard, a cross-sectional study in the UK and Australia showed that higher education was the most important factor associated with healthier dietary behavior in disadvantaged populations [39]. On the other hand, many unhealthy foods are cheap and tasty [40]. Thus, in poor families, unhealthy food choices may be an attempt to satisfy children’s constant demand for the food especially when families have low nutritional knowledge. Sugary products and processed meats in the unhealthy dietary pattern of this study are such cheap and tasty foods.

Despite the above arguments, results of this study suggested that the selection of foods by participants from both food secure and insecure conditions was strongly influenced by factors such as taste and food preference rather than healthfulness of foods. In fact, the taste seemed to be the most important factor in food selection after family budget. Although food secure participants and their family head had higher levels of literacy, the literacy did not seem to greatly influence food choice. The presence of red meat in healthy pattern 1 indicates that participants who consumed it had little concern about healthiness of the foods they consumed. In fact, it is more likely that food secure participants who followed the healthy dietary pattern consumed foods according to their preference and considering the affordability of foods in their budget rather than healthiness of foods [13]. Likewise, the existence of high-fat dairy in the unhealthy dietary pattern indicates that those who consumed it had no concern about food health otherwise they would have replaced high-fat dairy with low-fat dairy, especially when we consider that they cost the same in Iran.

### Food insecurity and eating habits

Food insecurity had associations with eating habits. Food insecure women consumed meals and snack less frequently than food secure women. In particular, food insecure participants ate less frequent breakfast and skipped it almost half of days. This finding is consistent with previous reports that have shown the habit of skipping breakfast in food insecure youth and adults [18, 41–43]. Breakfast is considered the most important meal of the day which has a good impact on diet quality and intake of essential nutrients [44]. Skipping breakfast is associated with failure in job and academic performance on one side and tendency towards ingestion of nutritionally poor foods in the midmorning on the other. Moreover, systematic reviews and meta-analyses of prospective cohort studies have shown that skipping breakfast is associated with increased risk of overweight and obesity [45], type 2 diabetes [46], cardiovascular diseases, and mortality [47], diseases that have a link with food insecurity [48]. Thus, in regions with high prevalence of food insecurity, school breakfast programs may help improve diet quality and reduce metabolic risks [49].

Similar to the breakfast, snacks also contribute to daily energy and nutrient intake [50]. However, depending on the type of snack (fruits and nuts vs. salty, sugary, and energy-dense snacks), snack consumption may be considered as a healthy or unhealthy dietary habit. Previous studies have reported conflicting results regarding snack consumption in food insecure individuals. For instance, in agreement with our results, Gedeon et al. reported that snacking habits was decreased in a sample of Lebanese children aged 5–11 years with severe food insecurity during COVID-19 pandemic [51]. In contrast, a study on 10 years old Portugal children indicated higher intake of salty snacks and soft drinks in higher levels of food insecurity [16]. Similarly, a qualitative study on American adults revealed that food insecure emerging and older adults were at a highest risk for frequent unhealthy snacking [52].

Although nutritional knowledge was not assessed, the higher rate of unhealthy cooking in food insecure participants suggests that at least a part of unhealthy dietary and eating habits in food insecure participants was due to the lack of knowledge and appropriate attitude in nutrition and eating practices. Rice, one of the two staple foods of Iranians (along with bread), is cooked in either pilaf or draining [28]. Draining is an unhealthy cooking procedure because rice nutrients are thrown away in draining while they are preserved in pilaf. Food insecure participants reported a higher rate of draining for rice cooking compared to food secure individuals, indicating their low knowledge and attitude towards the proper procedure of rice cooking. Likewise, frying, which is an unhealthy cooking procedure, was used more commonly by food insecure women compared to boiling and steaming. Drinking tea along or immediately after meals is a common unhealthy eating habits among Iranians [28]. Again, food insecure participants reported drinking tea around meals more often than food secure individuals. The higher prevalence of unhealthy eating habits among food insecure participants suggests their lower nutritional knowledge and inappropriate attitude in cooking when compared to food secure counterparts. Unfortunately, nutritional knowledge was not assessed in this study, but participants especially those with food insecurity had low education levels, and evidence indicates that people in low levels of literacy generally have poor nutritional knowledge [53, 54]. Previous studies have highlighted the inverse association between food insecurity and food/nutrition literacy [55, 56] or between financial status and nutrition literacy [53]. Future investigations need to assess the contribution of nutrition literacy/illiteracy in food selection and diet quality of food insecure individuals. In addition, the role that food shopping and preparation skills can play in reducing the impact of limited food budget on diet quality deserves evaluation. Moreover, it needs to be explored the extent to which educational interventions can mitigate adverse effects of food insecurity on diet quality.

### Strengths and limitations

This study was the first to examine the association of food insecurity and dietary patterns, cooking, and eating habits among Iranians. Identification of dietary patterns with “a posteriori” approach allowed us to examine the relationship of food insecurity with both healthy and unhealthy dietary patterns. Moreover, the association of food insecurity with cooking and eating habits had not been explored before. Semi-quantitative nature of FFQ allowed us to quantitatively evaluate the amount of foods and nutrients consumed, and the recalling foods consumed during the past year made it possible to extract existing dietary patterns. However, recalling foods eaten during the past year was subject to recall bias. Moreover, the participants were women, and thus a comparison could not be performed between men and women. In addition, the contribution of participants’ food and nutritional knowledge in diet planning and food choice was unclear. We, also, did not assess psychological problems such as depression and stress, which may have affected diet quality and eating habits. At last, participants were clients of primary healthcare centers, and hence the results may not be generalizable to all Iranian women. Besides, convenience sampling was a non-probability sampling method and subject to selection bias. Future research need to take these points into consideration.

## Conclusions

Overall, this study showed that food insecurity was associated with less healthy dietary patterns, lower intakes of fruit and vegetables, low-fat dairy, meats, legumes, and nuts, and higher consumption of processed meats. Food insecure women also reported less healthy eating and cooking habits. Future investigations are needed to find whether nutrition education programs are able to weaken the association of food insecurity with diet quality and eating habits.

### Electronic supplementary material

Below is the link to the electronic supplementary material.

Supplementary Material 1

## Data Availability

Please contact the corresponding author for data requests.
