# Supplementary material for: Food insecurity is associated with low diet quality and unhealthy cooking and eating habits in Iranian women
Source: J Health Popul Nutr. 2024 Mar 14;43:42. doi: 10.1186/s41043-024-00533-3 (PMC10941397; doi:10.1186/s41043-024-00533-3)
Supplement: Supplementary file 1 — Supplementary Material 1 [file 41043_2024_533_MOESM1_ESM.docx]

| Food groups | Number of missing values |
| --- | --- |
| Fruits | 13 |
| Red meat | 0 |
| Nuts | 1 |
| Legumes | 2 |
| Processed meats | 0 |
| Fish | 2 |
| Chicken | 0 |
| Low-fat dairy | 0 |
| Non-starchy vegetables | 21 |
| Refined grains | 4 |
| Potato | 1 |
| High-fat dairy | 0 |
| Sugary products | 13 |
| Egg | 0 |

Supplemental Table 1- The number of missing data in food groups that were imputed using multiple imputation method
